# Patient characteristics, comorbidities, and medication use for children with ADHD with and without a co-occurring reading disorder: A retrospective cohort study

**DOI:** 10.1186/1753-2000-5-38

**Published:** 2011-12-06

**Authors:** Peter M Classi, Trong K Le, Sarah Ward, Joseph Johnston

**Affiliations:** 1 Eli Lilly and Company, Lilly Corporate Center, Indianapolis, Indiana 46285 USA

**Keywords:** ADHD, Reading Disorder, medication use, comorbidities, patient characteristics

## Abstract

**Background:**

Children and adolescents with attention-deficit/hyperactivity disorder (ADHD) often have a co-occurring reading disorder (RD). The purpose of this research was to assess differences between children with ADHD without RD (ADHD-only) and those with ADHD and co-occurring RD (ADHD+RD).

**Methods:**

Using data from the U.S. Thomson Reuter Marketscan^® ^Databases for the years 2005 through 2007, this analysis compared the medical records--including patient demographics, comorbidities, and medication use--of children (age < 18) with ADHD-only to those with ADHD+RD.

**Results:**

Patients with ADHD+RD were significantly younger, more likely to have received a procedure code associated with formal psychological or non-psychological testing, and more likely to have been diagnosed with comorbid bipolar disorder, conduct disorder, or depression. They were no more likely to have received an antidepressant, anti-manic (bipolar), or antipsychotic, and were significantly less likely to have received a prescription for a stimulant medication.

**Conclusions:**

Relying on a claims database, there appear to be differences in the patient characteristics, comorbidities, and medication use when comparing children with ADHD-only to those with ADHD+RD.

## Background

The American Psychiatric Association's *Diagnostic and Statistical Manual of Mental Disorders, Fourth Edition *(DSM-IV), defines reading disorder (RD) as: "[R]eading achievement (i.e. reading accuracy, speed, or comprehension as measured by individually administered standardized tests) that falls substantially below that expected given the individual's chronological age, measured intelligence, and age-appropriate education [1]." While the rate of RD among all school-age children in the United States is an estimated 4% [2], up to nearly one-third (15%-30%) of children with a diagnosis of attention-deficit/hyperactivity disorder (ADHD) have a co-occurring diagnosis of RD [3,4]. Research has shown that children with ADHD and co-occurring RD (ADHD+RD) exhibit both the deficits in the basic semantics of language processing associated with RD and the higher-order executive function deficits of ADHD [5-11].

Given the fact that ADHD and RD often co-occur, a growing body of research has examined shared pathophysiological pathways of ADHD and RD. This research has shown that the two disorders share genetic and environmental factors [12,13], cognitive processes [14-16], aspects of brain anatomy and functioning [17], and treatment interventions [18]. These and other studies have also indicated that ADHD+RD may be a unique disorder with features not associated with either ADHD or RD in isolation. For example, children with ADHD and co-occurring reading or other learning disorders have certain social impairments not observed in "pure" ADHD or learning disorder groups, impairments that may lead to a greater likelihood of peer rejection or starting fights [19]. Still other literature indicates that those with ADHD and co-occurring RD or other learning disorders have more pervasive attention and visuomotor problems than those with either ADHD without learning disorders or learning disorders without ADHD [7].

The purpose of the present study was to better understand differences between ADHD patients with and without co-occurring RD, using two patient groups identified from a large managed care health care claims database. The primary objective was to compare these two groups on the basis of demographic characteristics, other comorbid disorders, and pharmacological treatments patterns. A secondary objective -- recognizing that use of medical billing records is a suboptimal means of identifying children with RD -- was to examine the prevalence of a coded diagnosis RD among ADHD patients and to compare this prevalence with other estimates of RD prevalence among ADHD patients from the literature.

## Methods

Data for this study came from the U.S. Thomson Reuters Marketscan^® ^Research Databases. These retrospective, claims databases are fully compliant with the Health Insurance and Portability Act (HIPAA) and capture person-specific clinical utilization, expenditures, and enrollment across inpatient, outpatient, prescription drug and carve-out services from a selection of large employers, health plans, and government and public organizations. The databases link paid claims and encounter data to detailed patient information across sites and types of providers over time and include private sector health data from approximately 100 payers and more than 500 million claim records. Data examined for this study spanned the years 2005 through 2007.

To be included in this study an individual had to be identified as having either ADHD-only or ADHD+ RD. In both groups, patients had to be diagnosed with ADHD based upon the receipt of an ICD-9-CM diagnostic code of 314.00 or 314.01 in the 2006 calendar year, with the first such date identified as the index date. In addition, individuals were required to be younger than 18 at index date and to have continuous insurance coverage, including prescription benefit coverage from 12 months prior to the index date (i.e., the pre-period) through 12 months post index date (i.e., the post-period). To enhance comparability between the ADHD only and ADHD+RD cohorts, all children with a diagnosis of mental retardation (ICD-9-CM of 317.xx, 318.xx, and 319.xx), pervasive development disorder (ICD-9-CM of 299.xx), or developmental delays (ICD-9-CM of 315.4x, 315.5x, 315.8x, or 315.9x) at any time from the start of the pre-period through the end of the post-period were excluded, as these diagnoses generally preclude or complicate a diagnosis of RD.

Given the above criteria, patients were then categorized as ADHD-only or ADHD+RD. In the ADHD-only cohort, patients were excluded if they had a diagnosis of RD (based upon receipt of an ICD-9-CM diagnostic code of 315.0x) at any time from the start of the pre-period through the end of the post-period. A total of 97, 703 children met the criteria for inclusion in the ADHD-only cohort.

For inclusion in the ADHD+RD cohort, individuals had to be diagnosed with ADHD and RD during the 2006 calendar year, with the first date of either diagnosis identified as the index date. Patients with an index diagnosis of ADHD were required to have at least one diagnosis of RD over the time period from the start of the pre-period through the end of the post-period. Similarly, those whose index diagnosis was for RD were required to have at least one diagnosis of ADHD from the start of the pre-period through the end of the post period. A total of 265 individuals were included in the ADHD+RD cohort.

The analysis compared patient demographics, comorbidities, and medication use between individuals with ADHD-only and those with ADHD+RD. Patient demographics included age, sex, region of residence, type of insurance coverage, and type of ADHD (with or without hyperactivity), while comorbid conditions included a variety of neuropsychiatric and behavioral conditions. The analysis also examined both medication use and length of therapy for ADHD medications (long-acting stimulants, short-acting stimulants, and non-stimulants) and other classes of medication used to treat psychiatric disorders (anti-depressants, anti-manic agents, antipsychotics, and anxiolytics). Differences in continuous variables were examined using t-statistics, while differences in categorical variables were examined using chi-square statistics. All analyses were conducted using SAS, version 9.1, and findings of P values of < 0.05 were considered statistically significant. As analyses were exploratory in nature, no adjustment for multiple comparisons was undertaken.

## Results

In this retrospective claims database, 0.27% of patients with ADHD were found to have a co-occurring RD. Patients in the ADHD-only cohort were older than those in the ADHD+RD cohort. For example, in the ADHD-only cohort, 49.1% of individuals were age 6-11 and 44.0% were age 12-17, while in the ADHD+RD cohort, these percentages were 63.8% and 33.6%, respectively. The majority of patients in both cohorts were male (71.4% and 67.5%), insured via a PPO (30.4% and 30.6%) or an HMO (30.6% and 32.8%), and diagnosed with ADHD with hyperactivity (70.5% and 70.9%). Patients in the ADHD+RD cohort were more likely to have received formal psychological testing (24.5% v 7.8%) or neuropsychological testing (5.7% v 0.9%) in a setting that is associated with insurance reimbursement, compared to patients with ADHD-only (see Table 1).

**Table 1:** Demographics and Patient Characteristics

Variable	ADHD Only (N = 97, 703)		ADHD + RD (N = 265)	
	
	N	%	N	%
Age*				

0-5	6708	6.9	7	2.6

6-11	47970	49.1	169	63.8

12-17	43025	44.0	89	33.6

Sex				

Male	69735	71.4	179	67.5

Female	27968	28.6	86	32.5

Insurance Type				

Comprehensive	23216	23.8	63	23.8

Health Maintenance Organization (HMO)	29880	30.6	87	32.8

Point of Service (POS)	13715	8.4	31	11.7

Preferred Provider Organization (PPO)	29730	30.4	81	30.6

Consumer-Driven Health Plan	685	0.7	1	0.4

Missing/Unknown	477	0.5	2	0.8

U.S. Region				

Northeast	5519	5.6	20	7.5

North Central	16394	16.8	32	12.1

South	26466	27.1	83	31.3

West	9357	9.6	16	6.0

Unknown	39967	40.9	114	43.0

ADHD Diagnosis Type**				

ADHD Without Mention of Hyperactivity	28830	29.5	77	29.1

ADHD With Hyperactivity	68873	70.5	188	70.9

Testing***				

Psychological Testing	7665	7.8	65	24.5

Neuropsychological Testing	873	0.9	15	5.7

* Age measured at index date. Mean age in the ADHD cohort was 10.8 years (std dev = 3.5 years; median = 11 years). Mean age in the ADHD + RD cohort was 10.2 years (std dev = 3.2 years; median = 10 years).

** Index ADHD, first ADHD in post period, or prior period diagnosis closest to the index date, in that order.

***Psychological and Neuropsychological Testing are from prior and post periods.

Comparing comorbid conditions (at the three digit ICD-9-CM level) revealed that individuals with ADHD+RD, compared to those with ADHD-only, were more likely to be diagnosed with bipolar disorder (9.4% v 6.4%; P = 0.043), conduct disorder (15.5% v 11.2%; P = 0.030), depression (14.3% v 9.9%; P < 0.001), and other learning disorders (12.8% v 3.3%; P < 0.001). Examining subcategories of these comorbidities (e.g., the four digit ICD-9-CM level) revealed that children with ADHD+RD were more likely to be diagnosed with oppositional-defiant disorder (11.7% v 8.0%; P = 0.028), major depressive disorder, recurrent episode (5.3% v 3.0%; P = 0.026), and the learning disorder of developmental speech or language disorder (7.9% v 2.2%; P < 0.0001). There was no difference in the two cohorts with regard to frequency of anxiety disorders, psychotic disorders, seizure disorders, or sleep disorders (see Table 2).

**Table 2:** Selected Comorbidities/Conditions

Comorbidity	ADHD		ADHD + RD		P Value
		
	N	%	N	%	
Anxiety Disorders	1227	1.3	4	1.5	0.580

Bipolar/Mania	6244	6.4	25	9.4	0.043

Conduct Disturbance	10984	11.2	41	15.5	0.030

Depression	9653	9.9	38	14.3	0.015

Learning Disorders	3251	3.3	34	12.8	< 0.001

Eating Disorders	134	0.1	1	0.4	0.306

Personality Disorders	120	0.1	0	0.0	1.000

Psychotic Disorders	869	0.9	5	1.9	0.085

Seizure Disorders	1097	1.1	5	1.9	0.239

Sleep Disorders	1866	1.9	1	0.4	0.069

Tics/Tourette's	689	0.7	0	0.0	0.272

All comorbidities examined from index date through end of post-period.

Based upon univariate analyses with chi-square tests for variables with counts of at least 5 and Fischer's exact test if any cell had less than 5 observations.

The two most commonly prescribed stimulant medications were extended release forms of amphetamine mixed salts (Adderall XR) and methylphenidate (Concerta), while atypical antipsychotics and antidepressants were the non-ADHD psychiatric medications most commonly prescribed. Relative to those with ADHD-only, children with ADHD+RD were significantly less likely to have received any stimulant medication (71.7% v 77.1%; P = 0.036). They were less likely, in particular, to have received the long-acting stimulant (Adderall XR) (23.0% v 31.6%; P = 0.003) or a short-acting amphetamine (4.9% v 9.5%; P = 0.011). There were no differences between the two cohorts with regard to the frequency of prescribing of anti-depressants, anti-manic agents, antipsychotics, or anxiolytics (see Table 3). While the analyses revealed significant differences in the frequency of prescriptions for stimulant medications for children with ADHD+RD compared to those with ADHD-only, among those prescribed such medication there was no difference in average length of prescription over the 12 month post period. Specifically, children with ADHD-only who were prescribed a stimulant received, on average, 6.5 months of therapy, compared to an average of 6.7 months for those with ADHD+RD (P = 0.609). Similarly, there were no differences among users of medications between the two cohorts with regard to average length of therapy for non-stimulants, antidepressants, antipsychotics, or anxiolytics (see Table 4).

**Table 3:** Medication Use

ADHD Medication	ADHD		ADHD + RD		P Value
		
	N	%	N	%	
Long-Acting Stimulants					

Adderall XR	30901	31.6	61	23.0	0.003

Concerta	30976	31.7	86	32.5	0.794

Daytrana	2749	2.8	11	4.2	0.189

Focalin XR	8002	8.2	30	11.3	0.064

Metadate CD	5426	5.6	19	7.2	0.251

Metadate ER	193	0.2	0	0.0	1.000

Methylin ER	1083	1.1	5	1.9	0.227

Methylphenidate HCL ER	0	0.0	0	0.0	N/A

Methylphenidate HCL SR	0	0.0	0	0.0	N/A

Ritalin LA	4574	4.7	15	5.7	0.451

Ritalin SR	39	0.0	0	0.0	1.000

Vyvanse	262	0.3	0	0.0	1.000

*Total Long-Acting*	69623	71.3	181	68.3	0.288

Short-Acting Stimulants					

Adderall	381	0.4	1	0.4	1.000

Amphetamine Salt Combo	9289	9.5	13	4.9	0.011

Amphetamine/Dextroamph	0	0.0	0	0.0	N/A

Cylert	0	0.0	0	0.0	N/A

Desoxyn	11	0.0	0	0.0	1.000

Dexedrine	27	0.0	0	0.0	1.000

Dexmethylphenidate HCL	109	0.1	1	0.4	0.258

Dextroamphetamine Sulfate	1231	1.3	1	0.4	0.272

Dextrostat	48	0.0	1	0.4	0.124

Dexampex	0	0.0	0	0.0	N/A

Focalin	3098	3.2	11	4.2	0.363

Methamphetamine HCL	0	0.0	0	0.0	N/A

Methylin	4986	5.1	13	4.9	0.884

Methylphenidate HCL	6701	6.9	19	7.2	0.841

Methylphenidate Hcl SA	0	0.0	0	0.0	N/A

Pemoline	10	0.0	0	0.0	1.000

Ritalin	397	0.4	2	0.8	0.293

Provigil	234	0.2	0	0.0	1.000

*Total Short-Acting*	22739	23.3	54	20.4	0.265

Total Stimulants	75354	77.1	190	71.7	0.036

Non-Stimulants					

Atomoxetine	13732	14.1	34	12.8	0.567

Bupropion	3486	3.6	10	3.8	0.857

Alpha 2 Agonists	11296	11.6	32	12.1	0.794

*Total Non-Stimulants*	25506	26.1	65	24.5	0.559

**Medications used to treat Other Mental Health Conditions**	**ADHD**		**ADHD + RD**		**P Value**
		
	N	%	N	%	

Anti-depressants					

MAOI's	0	0.0	0	0.0	N/A

Tricyclics	1734	1.8	3	1.1	0.638

SSRI's	9508	9.7	26	9.8	0.965

SNRI's	772	0.8	1	0.4	0.729

Other	5105	5.2	18	6.8	0.252

*Total Antidepressants*	14966	15.3	43	16.2	0.682

Antimanic (bipolar) agents	7208	7.4	21	7.9	0.734

Antipsychotics					

Typical	444	0.5	0	0.0	0.638

Atypical	15593	16.0	52	19.6	0.104

*Total Antipsychotics*	15703	16.1	52	19.6	0.116

Anxiolytics					

Benzodiazepines	1807	1.8	3	1.1	0.643

Others	2388	2.4	5	1.9	0.557

*Total Anxiolytics*	3982	4.1	8	3.0	0.385

All comorbidties examined from index date through end of post-period.

Based upon univariate analyses with chi-square tests for variables with counts of at least 5 and Fischer's exact test if any cell had less than 5 observations.

**Table 4:** Length of Therapy-By Medication Class

Medication Class	ADHD				ADHD + RD				P Value
		
	N	Mean (Months)	SD	Median (Months)	N	Mean (Months)	SD	Median (Months)	
Long-Acting Stimulants	69623	6.5	3.57	6.5	181	6.4	3.36	6.3	0.722

Short-Acting Stimulants	22739	4.1	3.28	3.0	54	3.2	2.88	2.0	0.053

Any Stimulant	75354	6.7	3.52	6.8	190	6.5	3.33	6.9	0.609

Non-Stimulants	25506	5.8	3.80	5.6	65	5.2	3.53	4.8	0.212

Antidepressants	14966	5.4	3.78	4.8	43	4.8	3.88	3.0	0.303

Antipsychotics	15703	6.4	3.75	6.7	52	6.3	3.67	7.3	0.863

Anxiolytics	3982	1.9	2.83	0.9	8	1.4	1.64	0.8	0.578

N is the total number of patients with non-missing days supply.

T-tests were used to examine differences in mean length of therapy between the two groups.

## Discussion

In contrast to the literature that suggests that RD co-occurs in 15-30% of children with ADHD [3,4], less than 1% of children with ADHD in our study cohort were found to have a co-occurring coded diagnosis of RD. This almost certainly reflects the incomplete ascertainment of reading disorders using administrative claims data and highlights the fact that claims data can provide valid epidemiologic data only to the extent that the conditions of interest are diagnosed, managed and reimbursed in traditional medical settings. Learning disorders, including reading disorders, are typically recognized and managed in an educational rather than a medical setting, and formal assessment typically involves the services of providers not routinely covered by traditional medical insurance (i.e., educational or clinical psychologists and psychometrists, rather than psychiatrists and other physician providers). In contrast, ADHD, while often first recognized at home or in the classroom, is commonly diagnosed by physicians, at least in part because of the availability of effective pharmacological treatment options [20]. Consequently, it should be recognized that our results pertain to a select subset of all children with ADHD and RD, and our findings should be interpreted accordingly.

Despite this caveat, results of this analysis suggest that children with ADHD+RD may differ from those with ADHD alone in several important respects. First, a greater proportion of the ADHD+RD children were 6-11 years old relative to 12-17 years old (63.8% v 33.6%). In contrast, the ADHD-only children were nearly equally distributed in the 6-11 year old and 12-17 year old groups (49.1% v 44.0%). This difference in age distribution may reflect the fact that RD is typically identified when reading instruction begins in school, i.e., either the end of kindergarten or the beginning of first grade [1,21], whereas ADHD requires the identification of symptoms in multiple domains of functioning (e.g., home, school) before it can be diagnosed [1]. Therefore, as the DSM-IV states: "[M]any individuals are diagnosed [with ADHD] after the symptoms have been present for a number of years, especially in the case of individuals with Predominantly Inattentive Type [1]." Alternatively, this age discrepancy could be a function of the non-representativeness of our ADHD+RD cohort. While we did exclude children with mental retardation, pervasive developmental disorder and other specific developmental delays, it may be the case that our ADHD+RD cohort included children with multiple and/or more complex disorders, a subset of children more likely to come to medical attention at an earlier age.

A large body of literature has provided evidence of a neurological basis for RD [22-34]. In the current study, a significantly higher percentage of ADHD+RD children relative to ADHD-only individuals had psychological testing (24.5% v 7.8%) and neuropsychological testing (15% v 0.9%). This finding reflects the following recent statement from the American Academy of Pediatrics et al. that: "Reading involves the integration of multiple factors related to a person's experience, ability, and neurologic functioning.... There is solid scientific evidence that supports the neurologic basis for the phonological coding deficit theory of reading disabilities [35]." Including psychological or neuropsychological testing in the process of diagnosing RD is compliant with current medical guidelines, which state that, "Children with learning disabilities should undergo assessments of their health, development, hearing, and vision and, when appropriate, medical and psychological interventions for associated and related treatable conditions [36]."

This study also revealed significant differences between the ADHD+RD and ADHD-only cohorts relative to comorbidities and medication use. Consistent with previous research showing a strong association between ADHD+RD and antisocial behavioral disorders, including aggression, delinquency, oppositional defiant disorder [34,37,38], as well as between RD and internalizing psychiatric disorders, such as depression [34], the ADHD+RD cohort in this study had a higher rate of comorbid illness, including bipolar/mania, conduct disturbance, oppositional defiant disorder, depression, and learning disorders (see Table 2). Parents, educators, and physicians should be watchful for signs of these disorders in children with both ADHD and RD.

Notably, although the patients with ADHD+RD in this study were more likely to be diagnosed with depression, bipolar/mania, or conduct disorder, they were no more likely to be prescribed antidepressant, anti-manic (bipolar), antipsychotic, or any other type of medication. Neither were those with ADHD+RD more likely to be prescribed any of the following non-stimulant medications used in the treatment of ADHD: atomoxetine [39,40], tricyclic antidepressants [41], or bupropion [42,43]. Moreover, relative to the ADHD-only group, the children with ADHD+RD were *less *likely to receive stimulant medication, which is also commonly prescribed for the treatment of ADHD [43]. Although the ADHD+RD group were less likely to be prescribed stimulant medication, those who did receive any medication had the same mean length of therapy as those in the ADHD-only cohort taking that medication (see Table 4).

The findings of this study should be interpreted in the context of the limitations of the study design. First, the use of diagnostic codes to identify individuals is not as rigorous as formal diagnostic assessments for identifying people with ADHD or RD. Because RD may be diagnosed outside of a medical system that is associated with insurance reimbursement claims, use of such a claims database may preclude capturing a large segment of the ADHD+RD population. As mentioned above, this analysis found that less than 1% of individuals with ADHD received a diagnosis of RD, while the literature suggests that RD may co-occur in 15-30% of ADHD patients [3,4]. In addition, the study focused exclusively on patients with medical and prescription benefit coverage. Given these limitations, the results may not be generalizeable to other populations. Third, as this study was descriptive in nature, it did not control for the impact of differences in patient characteristics (e.g., age distribution, severity of RD or ADHD, etc.) between the two cohorts of children. This limitation, in turn, precluded any inferences about causality. For example, it is possible that the ADHD+RD cohort in this study were prescribed less stimulant medication relative to the ADHD-only cohort due to the fact that they were younger; however, this hypothesis was not testable given the study design.

Additionally, the number of unique physician visits was not captured in this analysis. While it appears that children with ADHD + RD present with more medical conditions and psychiatric comorbidities compared to children with ADHD alone, this may be due in part to the fact that children with ill-defined or complex developmental disorders are seen by multiple physicians and may receive multiple "rule out" diagnoses before receiving an accurate and comprehensive assessment of their condition. Finally, the use of medical claims data precludes the use of patient assessments; as a result, the analysis could not examine quality of life, functioning, or any clinical outcomes.

## Conclusions

This retrospective, descriptive analysis revealed significant differences between children with ADHD+RD and those with ADHD-only. In this study, the cohort with ADHD+RD had a higher burden of comorbidity, including a greater likelihood of a comorbid diagnosis of bipolar/mania, conduct disturbance, oppositional defiant disorder, depression, and learning disorders. At the same time, the children with ADHD+RD were no more likely to have been prescribed antidepressant, anti-manic (bipolar), antipsychotic, or any other type of medication, and were less likely to have been prescribed stimulant medication. These findings indicate the need for further research into the epidemiology, treatment and associated outcomes for children with ADHD+RD. They also indicate that the burden of ADHD+RD is unique and substantial. The special characteristics of ADHD+RD should be considered when conducting clinical evaluations and targeted treatment approaches. All results should be interpreted cautiously given the limited ability to ascertain RD using claims data, and future research into RD should focus on developing and using more broadly representative datasets.

## Competing interests

The authors declare that they have no competing interests.

## Authors' contributions

PC made substantial contributions to study conception, study design, interpretation of data, drafting and revising of manuscript and has read and given approval of the final version to be published. TL made substantial contributions to study design, data analyses, interpretation of data, drafting and revising of manuscript and has read and given approval of the final version to be published. SW made substantial contributions to study design, drafting and revising of manuscript and has read and given approval of the final version to be published. JJ made substantial contributions to study conception, study design, interpretation of data, drafting and revising of manuscript and has read and given approval of the final version to be published.

## References

[B1] American Psychiatric Association Diagnostic and Statistical Manual of Mental Disorders DSM-IV-TR Fourth Edition 2000 4 American Psychiatric Publishing, Inc

[B2] Sadock BJ Sadock VA Kaplan & Sadock's concise textbook of clinical psychiatry 2008 Lippincott Williams & Wilkins

[B3] Barkley RA Major life activity and health outcomes associated with attention-deficit/hyperactivity disorder J Clin Psychiatry 2002 63 Suppl 12 10 15 12562056

[B4] Tannock R Brown T Thomas E Brown Attention-deficit disorders with learning disorders in children and adolescents Attention-Deficit Disorders and Comorbidities in Children, Adolescents, and Adults 2000 Washington, DC: American Psychiatric Press 231 295

[B5] Douglas VI Benezra E Supraspan verbal memory in attention deficit disorder with hyperactivity normal and reading-disabled boys J Abnorm Child Psychol 1990 18 617 638 10.1007/BF01342751 2074344

[B6] Felton RH Wood FB Cognitive deficits in reading disability and attention deficit disorder J Learn Disabil 1989 22 3 13 22 10.1177/002221948902200102 2703785

[B7] Korkman M Pesonen AE A comparison of neuropsychological test profiles of children with attention deficit-hyperactivity disorder and/or learning disorder J Learn Disabil 1994 27 383 392 10.1177/002221949402700605 8051511

[B8] Nigg JT Hinshaw SP Carte ET Treuting JJ Neuropsychological correlates of childhood attention-deficit/hyperactivity disorder: explainable by comorbid disruptive behavior or reading problems? J Abnorm Psychol 1998 107 468 480 9715582 10.1037/0021-843X.107.3.468

[B9] Purvis KL Tannock R Language abilities in children with attention deficit hyperactivity disorder, reading disabilities, and normal controls J Abnorm Child Psychol 1997 25 133 144 10.1023/A:1025731529006 9109030

[B10] Willcutt EG Pennington BF Comorbidity of reading disability and attention-deficit/hyperactivity disorder: differences by gender and subtype J Learn Disabil 2000 33 179 191 10.1177/002221940003300206 15505947

[B11] Willcutt EG Pennington BF Boada R Ogline JS Tunick RA Chhabildas NA Olson RK A comparison of the cognitive deficits in reading disability and attention-deficit/hyperactivity disorder J Abnorm Psychol 2001 110 157 172 11261391 10.1037//0021-843x.110.1.157

[B12] Petryshen TL Pauls DL The genetics of reading disability Curr Psychiatry Rep 2009 11 149 155 10.1007/s11920-009-0023-z 19302769

[B13] Willcutt EG Pennington BF Comorbidity of Reading Disability and Attention-Deficit/Hyperactivity Disorder Journal of Learning Disabilities 2000 33 179 191 10.1177/002221940003300206 15505947

[B14] Shanahan MA Pennington BF Yerys BE Scott A Boada R Willcutt EG Olson RK DeFries JC Processing speed deficits in attention deficit/hyperactivity disorder and reading disability J Abnorm Child Psychol 2006 34 585 602 16850284 10.1007/s10802-006-9037-8

[B15] Tridas EQ Ed From ABC to ADHD: What parents should know about dyslexia andattention problems 2007 Baltimore: International Dyslexia Association

[B16] Willcutt EG Betjemann RS Pennington BF Olson RK Defries JC Wadsworth SJ Longitudinal Study of Reading Disability and Attention-Deficit/Hyperactivity Disorder: Implications for Education Mind, Brain, and Education 2007 1 181 192 10.1111/j.1751-228X.2007.00019.x

[B17] Eden GF Vaidya CJ ADHD and developmental dyslexia: two pathways leading to impaired learning Ann N Y Acad Sci 2008 1145 316 327 10.1196/annals.1416.022 19076406

[B18] Bental B Tirosh E The Effects of Methylphenidate on Word Decoding Accuracy in Boys With Attention-Deficit/Hyperactivity Disorder Journal of Clinical Psychopharmacology 2008 28 89 92 10.1097/jcp.0b013e3181603f0e 18204348

[B19] Flicek M Social status of boys with both academic problems and attention-deficit hyperactivity disorder J Abnorm Child Psychol 1992 20 353 366 10.1007/BF00918981 1527277

[B20] LD OnLine: Who Can Diagnose LD and/or ADHD http://www.ldonline.org/article/6027

[B21] Developmental reading disorder | Encyclopedia of Psychology | Find Articles at BNET http://findarticles.com/p/articles/mi_g2699/is_0004/ai_2699000441/

[B22] Cao F Bitan T Chou T-L Burman DD Booth JR Deficient orthographic and phonological representations in children with dyslexia revealed by brain activation patterns J Child Psychol Psychiatry 2006 47 1041 1050 10.1111/j.1469-7610.2006.01684.x 17073983 PMC2617739

[B23] Eden GF Zeffiro TA Neural systems affected in developmental dyslexia revealed by functional neuroimaging Neuron 1998 21 279 282 10.1016/S0896-6273(00)80537-1 9728909

[B24] Hynd GW Semrud-Clikeman M Lorys AR Novey ES Eliopulos D Brain Morphology in Developmental Dyslexia and Attention Deficit Disorder/Hyperactivity Arch Neurol 1990 47 919 926 10.1001/archneur.1990.00530080107018 2375699

[B25] Petersen SE Fox PT Posner MI Mintun M Raichle ME Positron emission tomographic studies of the cortical anatomy of single-word processing Nature 1988 331 585 589 10.1038/331585a0 3277066

[B26] Pugh KR Mencl WE Jenner AR Katz L Frost SJ Lee JR Shaywitz SE Shaywitz BA Functional neuroimaging studies of reading and reading disability (developmental dyslexia) Ment Retard Dev Disabil Res Rev 2000 6 207 213 10.1002/1098-2779(2000)6:3<207::AID-MRDD8>3.0.CO;2-P 10982498

[B27] Pugh KR Mencl WE Jenner AR Katz L Frost SJ Lee JR Shaywitz SE Shaywitz BA Neurobiological studies of reading and reading disability J Commun Disord 2001 34 479 492 10.1016/S0021-9924(01)00060-0 11725860

[B28] Shaywitz BA Shaywitz SE Blachman BA Pugh KR Fulbright RK Skudlarski P Mencl WE Constable RT Holahan JM Marchione KE Fletcher JM Lyon GR Gore JC Development of left occipitotemporal systems for skilled reading in children after a phonologically- based intervention Biol Psychiatry 2004 55 926 933 10.1016/j.biopsych.2003.12.019 15110736

[B29] Shaywitz BA Shaywitz SE Pugh KR Mencl WE Fulbright RK Skudlarski P Constable RT Marchione KE Fletcher JM Lyon GR Gore JC Disruption of posterior brain systems for reading in children with developmental dyslexia Biol Psychiatry 2002 52 101 110 10.1016/S0006-3223(02)01365-3 12114001

[B30] Shaywitz SE Shaywitz BA Fulbright RK Skudlarski P Mencl WE Constable RT Pugh KR Holahan JM Marchione KE Fletcher JM Lyon GR Gore JC Neural systems for compensation and persistence: young adult outcome of childhood reading disability Biol Psychiatry 2003 54 25 33 10.1016/S0006-3223(02)01836-X 12842305

[B31] Silani G Frith U Demonet J-F Fazio F Perani D Price C Frith CD Paulesu E Brain abnormalities underlying altered activation in dyslexia: a voxel based morphometry study Brain 2005 128 2453 2461 10.1093/brain/awh579 15975942

[B32] Temple E Poldrack RA Salidis J Deutsch GK Tallal P Merzenich MM Gabrieli JD Disrupted neural responses to phonological and orthographic processing in dyslexic children: an fMRI study Neuroreport 2001 12 299 307 10.1097/00001756-200102120-00024 11209939

[B33] Temple E Deutsch GK Poldrack RA Miller SL Tallal P Merzenich MM Gabrieli JDE Neural deficits in children with dyslexia ameliorated by behavioral remediation: Evidence from functional MRI Proceedings of the National Academy of Sciences of the United States of America 2003 100 2860 2865 10.1073/pnas.0030098100 12604786 PMC151431

[B34] Willcutt EG Pennington BF Psychiatric comorbidity in children and adolescents with reading disability J Child Psychol Psychiatry 2000 41 1039 1048 10.1111/1469-7610.00691 11099120

[B35] American Academy of Pediatrics S on O American Academy of Ophthalmology undefined American Association for Pediatric Ophthalmology and Strabismus American Association of Certified Orthoptists Learning Disabilities, Dyslexia, and Vision Pediatrics 2009 124 837 844 19651597 10.1542/peds.2009-1445

[B36] Committee on Children With Disabilities The Pediatrician's Role in Development and Implementation of an Individual Education Plan (IEP) and/or an Individual Family Service Plan (IFSP) Pediatrics 1999 104 124 127 10390275 10.1542/peds.104.1.124

[B37] Frick PJ Kamphaus RW Lahey BB Loeber R Christ MA Hart EL Tannenbaum LE Academic underachievement and the disruptive behavior disorders J Consult Clin Psychol 1991 59 289 294 2030190 10.1037/0022-006X.59.2.289

[B38] Maughan B Pickles A Hagell A Rutter M Yule W Reading problems and antisocial behaviour: developmental trends in comorbidity J Child Psychol Psychiatry 1996 37 405 418 10.1111/j.1469-7610.1996.tb01421.x 8735440

[B39] Kratochvil CJ Heiligenstein JH Dittmann R Spencer TJ Biederman J Wernicke J Newcorn JH Casat C Milton D Michelson D Atomoxetine and methylphenidate treatment in children with ADHD: a prospective, randomized, open-label trial J Am Acad Child Adolesc Psychiatry 2002 41 776 784 10.1097/00004583-200207000-00008 12108801

[B40] Michelson D Adler L Spencer T Reimherr FW West SA Allen AJ Kelsey D Wernicke J Dietrich A Milton D Atomoxetine in adults with ADHD: two randomized, placebo-controlled studies Biol Psychiatry 2003 53 112 120 10.1016/S0006-3223(02)01671-2 12547466

[B41] Jadad AR Boyle M Cunningham C Kim M Schachar R Treatment of attention-deficit/hyperactivity disorder Evid Rep Technol Assess (Summ) 1999 i viii 1-341 PMC4782276 10790990

[B42] Conners CK Casat CD Gualtieri CT Weller E Reader M Reiss A Weller RA Khayrallah M Ascher J Bupropion hydrochloride in attention deficit disorder with hyperactivity J Am Acad Child Adolesc Psychiatry 1996 35 1314 1321 10.1097/00004583-199610000-00018 8885585

[B43] Subcommittee on Attention-Deficit/Hyperactivity Disorder Clinical Practice Guideline: Treatment of the School-Aged Child With Attention-Deficit/Hyperactivity Disorder Pediatrics 2001 108 1033 1044 11581465 10.1542/peds.108.4.1033

